# Effects of A “Modified” Otago Exercise Program on the Functional Abilities and Social Participation of Older Adults Living in the Community—The AGA@4life Model

**DOI:** 10.3390/ijerph17041258

**Published:** 2020-02-15

**Authors:** Anabela Correia Martins, Daniela Guia, Marina Saraiva, Telmo Pereira

**Affiliations:** 1 Physiotherapy Department, Coimbra Health School, Polytechnic of Coimbra, 3046-854 Coimbra, Portugal; daniela_guia@hotmail.com (D.G.); marina.saraiva@outlook.com (M.S.); 2 Clinical Physiology Department, Coimbra Health School, Polytechnic of Coimbra, 3046-854 Coimbra, Portugal; telmo@estescoimbra.pt

**Keywords:** “modified” Otago Exercise Program, Exergames, older adults, falls, healthy ageing

## Abstract

Strength and balance exercises form part of multifactorial programs to reduce the risk of falling and promote active ageing. The aim of this study was to evaluate the effect of a strength and balance exercise program, adapted from the traditional Otago Exercise Program (OTAGO) into a technological system. A non-randomized experimental study enrolled 34 participants (83.24 ± 6.89 years) from a daycare center in Portugal, who were distributed into an intervention group (IG; 18 participants) and a control group (CG; 16 participants). The IG underwent a “modified” OTAGO incorporated in a technological system using pressure and inertial sensors, feedback, and Exergames for 8 weeks, 3 times a week. The CG continued their regular activities. Outcome measures were evaluated at baseline and after 8 weeks of intervention. After the program, differences were observed between the groups in handgrip strength (*p* = 0.03), step test (*p* = 0.03), 4stage balance test “modified” (*p* < 0.001) and activities and participation profile related to mobility (PAPM) (*p* < 0.001). The IG showed positive results in the self-efficacy for exercise (*p* = 0.03), PAPM (*p* = 0.00) and all functional tests, except for timed up and go (*p* = 0.35). No significant changes were observed in the CG. The results support this intervention program as a good exercise solution to improve functional abilities, social participation, and self-efficacy, reducing the risk of falling.

## 1. Introduction

Ageing is a major social and economic challenge in modern times. In Portugal, the percentage of adults over 65 years old in the population has increased from 16% in 2001, to 21.5% in 2017; further, 13.4% of that 21.5% are above 85 years old. Ageing is associated with a progressive decline in several physiological functions, and therefore has consequences in terms of overall functioning, including functional abilities and social participation, autonomy, risk of falls, and overall health; thus, the implementation of strategies to promote healthy ageing is paramount for modern societies [1].

Recently, in the Global Strategy and Action Plan for Ageing and Health [1], it was assumed that healthy ageing is relevant to all people. It is defined as the process of development and maintenance of functional ability that enables continued social participation and lifelong wellness. According to the same document, social participation is determined by the intrinsic ability of the individual person, environmental factors, and the interactions between the two.

Changes throughout the ageing process such as reduced physical activity levels and decreased gait speed and muscle strength are associated with an earlier or faster decline in mobility [2,3]. The incidence and prevalence of gait impairments are high in adults living in the community, which may increase their risk of falls, institutionalization, and mortality [4].

Balance impairment due to sensorimotor changes, the decrease or loss of proprioception, muscular strength, reaction time, visual and vestibular ability, or other pathological conditions, all increase the likelihood of falls and consequent injuries throughout the lifespan [5]. These falls, defined by the WHO as unexpected and unintentional episodes in which the individual rests inadvertently on the ground, floor, or another lower level [6], increase the likelihood of loss of autonomy, independence, and quality of life in older adults [7]. It is estimated that approximately 30% of individuals aged over 65 years suffer at least one episode of fall per year, and that the risk increases to 50% beyond the age of 80 [8,9]. Falls therefore constitute a major public health problem in this particular population group [10].

Decreased muscle strength due to the ageing process is also related to the risk of falls during gait. Considering the importance of gait in functioning, independence, and mobility [11,12], it is fundamentally important to prescribe exercise programs to improve strength [13].

Interventions to prevent falls should be multifactorial, and adjusted to the risk factors identified at the baseline evaluation. An exercise component including strength, balance, and gait training should always be included [14,15,16].

Exercise is a fundamental intervention tool in improving the functional ability and quality of life of a person throughout life [17]. There is strong scientific evidence supporting the efficacy of exercise in the prevention of falls in older adults. The recommended exercise programs for older adults include aerobic, strength, and balance exercises with the main objective of promoting functional abilities and preventing/controlling chronic diseases and falls [18]. The Otago Exercise Program (OTAGO) is commonly applied for such purposes. It is a program that incorporates moderate intensity strength exercises focusing on the lower limbs and balance [19], to be performed for about 30 min at least three times a week. Walking on alternate days at least twice a week can also be featured [20]. This type of program is significantly effective in increasing the balance, gait, and muscular strength of the lower limbs, consequently reducing the risk of falls in older adults [16,21].

The use of biofeedback provides individuals with additional information about their performance, allowing them to develop changes in their behavior or posture, which could potentially lead to better performance of the tasks. In the literature, evidence exists supporting the integration of visual biofeedback in the balance training programs, as this provides additional benefits compared to traditional exercise programs or no intervention [22].

The FallSensing Exergames, a wearable sensor-based exercise program based on OTAGO [23,24,25], was adopted by the Aga@4life project, which aimed to promote active and healthy ageing via a comprehensive geriatric approach, through the implementation of an integrated, multidisciplinary, and tailored intervention program in a cohort of older adults. This study aimed to evaluate the effect of a “modified” OTAGO, incorporated within a technological system, on functional abilities, social participation, and self-efficacy for exercise in older adults.

## 2. Materials and Methods

### 2.1. FallSensing Exergames

The FallSensing Exergames have been described in detail in previous literature [26] and include three mini-games to be played in teams. Each mini-game combines up to three different exercises from the original OTAGO. Players use a wearable inertial sensor to track their movement.

Mini-game 1 includes the knee bends and sit to stand exercises from the original OTAGO, monitored with a sensor worn on the thigh.

Mini-game 2 includes the side hip strengthening, the front knee strengthening, and the back knee strengthening exercises from the original OTAGO, with the sensor worn on the ankle.

Mini-game 3 includes the calf raises and toe raises exercises from the original OTAGO, with sensors worn on top of the foot.

**Figure 1:**
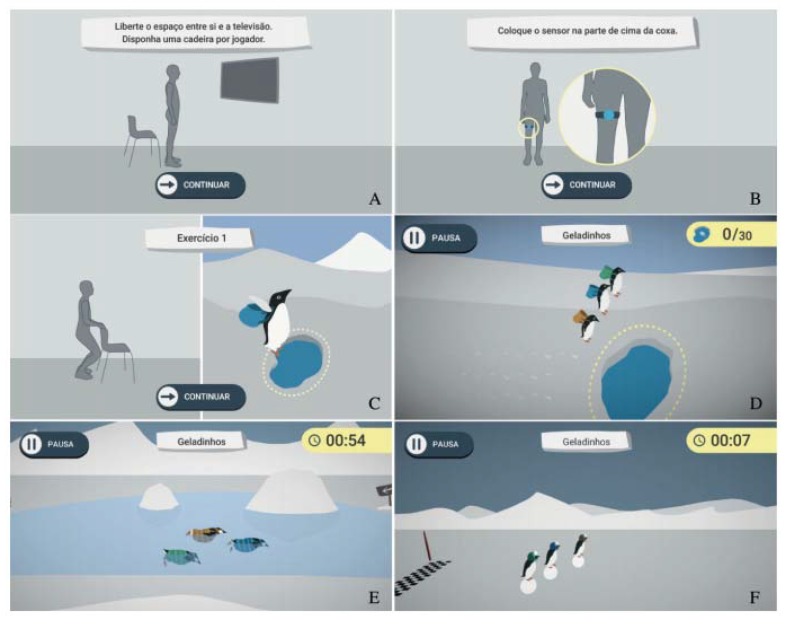
(**A**) Instructions on the space required to perform exercise; (**B**) Instructions on how to fix the sensor; (**C**) Exercise instructions; (**D**) Mini-game 1; (**E**) Mini-game 2; (**F**) Mini-game 3 (Source: [26]).

Figure 1 illustrates the Exergames layout.

### 2.2. Participants and Ethics

The participants were recruited from a day care center in Coimbra district, Portugal, where they spend part of the day. The inclusion criteria were as follows: Persons aged 65 years or over, female and male, physically autonomous, without a prior history of cerebrovascular, neurological disorders, or depression. Ethical approval was obtained from the Research Ethics Committee of the Polytechnic Institute of Coimbra (No.6/2017). Participants were given a written informed consent form before data collection as per the Declaration of Helsinki, and the study was registered in ClinicalTrials.gov with the identifier (NCT number): NCT03623919. The anonymity and confidentiality of the collected data were assured. The study was conducted for scientific purposes only, so there was no conflict of interest to be declared.

### 2.3. Procedure

The participants were enrolled to participate in a non-randomized experimental study in January 2018. During February and March 2018, a baseline multidisciplinary diagnostic evaluation of each participant was performed, comprising the gathering of relevant demographic and clinical information, including data on each individual’s comorbidities, ongoing treatments, diet and physical activity profile, cardiovascular risk profile, functional ability, fear of falling, and history of falls in the previous 12 months.

Thirty-four participants were divided in two groups according to their willingness to participate in in the study. The intervention group (IG) was subject to a tailored intervention program (*n* = 18), consisting of an exercise plan incorporated in a technological system using pressure and inertial sensors, feedback, and Exergames for 8 weeks, three times a week, lasting approximately 20 min on each occasion. The control group (CG) was encouraged to maintain their usual daily routines (outdoor aerobic exercise) (*n* = 16). The intensity of the exercises progresses depended on participant feedback and recommendations of the original program. After the eight weeks had ended, all the participants were re-assessed by repeating the initial protocol.

### 2.4. Outcomes

To measure the participants’ functioning, the protocol included the functional ability parameters the self-efficacy for exercise and the social participation profile, which were measured by the following tests and questionnaires:

#### 2.4.1. Handgrip Strength (HS)

Handgrip strength is an indicator of total muscular strength [27], as it is also correlated with the strength of the lower limbs [28]. A Jamar hydraulic hand dynamometer was used (Kg/f). The assessment was performed with individuals seated comfortably and with the dominant arm together with the body (without support), with the shoulder in adduction, the elbow flexed at 90°, and the forearm in a neutral position [29,30]. The individual was instructed to exert their maximal grip strength for five seconds, only once.

#### 2.4.2. 30 Seconds Sit-to-Stand (30s STS)

This assessment was used to evaluate the functional strength of the lower limbs. It consists of sitting on, and then standing up from, a chair, with arms crossed over the chest, as many times as possible in a period of 30 s. If the participant completes a rise of more than halfway up at the end of 30 s, this is counted as a full stand. The number of stands an individual can complete in 30 s are counted [31,32].

#### 2.4.3. Timed Up and Go (TUG) 

This test assesses mobility, balance, and risk of fall [33,34]. The individual is instructed to sit on a chair (height between 44 and 47 cm) [35] with his back well against its back [36], and is then asked to perform the task of getting up from the chair, walk three meters as fast as possible, turn, return towards the chair, and sit down again, with the time taken registered [34,37]. The test is performed only once [36].

#### 2.4.4. Step Test (ST)

This test was designed to assess dynamic standing balance [37,38]. The individual has to go up and down a step (7.5 cm height, 55 cm width, 35 cm depth) as many times as possible during a period of 15 s, always with the same foot. The test is interrupted if loss of balance occurs and the result is given as the number of repetitions performed during the 15 s [38,39,40].

#### 2.4.5. 4 Stage Balance Test “Modified” (4StageBTM)

This test consists of performing four different feet positions, with increased degrees of difficulty as the test progresses. The individual, with his arms along his body, barefoot and without support, has to hold each position for 10 s and only moves to the next position if there was no imbalance during that time [41,42]. The positions are as follows: feet together side by side, semi-tandem, tandem, and one-legged stance [41]. Each position was held both with eyes open and closed, with the exception of the last position (one legged stance), which was performed only with the eyes open. The sequence was side by side stance (eyes open); side by side stance (eyes closed); semi-tandem (eyes open), semi-tandem (eyes closed); tandem (eyes open); tandem (eyes closed), and one leg stance (eyes open). Scores were recorded corresponding to the number of positions performed successfully [31].

#### 2.4.6. Self-Efficacy for Exercise (SEE)

This test assesses the confidence that the individual has in his/her ability to perform the exercise. It is a 5-item scale that analyzes the confidence an individual presents in performing physical exercise. The questionnaire was administered by interview; each of these items was graduated with a 5-point Likert scale, being defined as 1 “not at all true”, 2 “slightly true”, 3 “moderately true”, or 4 “completely true”. The total score was taken as the sum of the scores of each item, varying between 5 and 20. The SEE internal consistency, measured by the Cronbach’s alpha, was 0.86 [43].

#### 2.4.7. Activities and Participation Profile related to Mobility (PAPM)

The PAPM evaluates difficulties in carrying out daily living activities such as interactions and social relations, education, employment, money management, and community and social life. It consists of 18 items, quoted from 0 to 4, where 0 represents “no limitation or restriction”, 1 “mild limitation or restriction”, 2 “moderate limitation or restriction”, 3 “severe limitation or restriction”, 4 “complete limitation or restriction”, and NA stands for “not applicable”. The total score was obtained through the quotient between the sum of the score obtained in each item answered and the number of items answered (0–4). The PAPM internal consistency, measured by Cronbach’s alpha, was 0.90 [44].

### 2.5. Statistical Analysis

The data was gathered in Excel 2016 (Microsoft Office, Redmond, WA, USA), and imported to SPSS version 24.0 (IBM, Armonk, NY, USA) for statistical analysis.

Categorical variables were reported as frequencies and percentages, and χ2 or Fisher exact tests were used when appropriate. The Shapiro–Wilks test was used to confirm the normal distribution of all continuous variables, expressed as mean and standard deviation (SD). Student’s t test was applied for baseline group comparisons. Individual variables were checked for homogeneity of variance using Levene’s test. A 2-factor mixed-design ANOVA was used to evaluate the modifications of variables between the baseline and the post-intervention evaluation in each group, and between groups. The Greenhouse–Geisser correction was used when sphericity was violated, and the Bonferroni adjustment was adopted for multiple comparisons designed to identify the significant effects of a factor. For between-groups comparison, an additional ANCOVA was performed over the post-intervention data, adjusting for the baseline data (entered as a covariate into the model). A *p* < 0.05 was considered significant. The magnitude of the effects was also checked with the η2 value.

## 3. Results

The demographic characteristics of the 34 participants are described in Table 1. The mean age was 83.24 ± 6.89 years (76.50% females). Most of the participants self-reported a fear of falling (85.30%), 41.20% referred to a fall history in the previous 12 months, and 76.5% presented a sedentary lifestyle. To stand from a chair, 73.53% of the participants reported needing upper extremities assistance.

The CG and IG were quite homogenous at the baseline features, with no statistically significant differences observed in terms of age, gender, history of fall, fear of falling, or sedentary lifestyle. 

The program was completed by all participants, with an adherence rate of 100%. Regarding the functional parameters at baseline, no significant differences were observed between groups in terms of HS (*p* = 0.16), 30s STS (*p* = 0.16), ST (*p* = 0.13), and 4StageBTM (*p* = 0.06). Significant baseline differences were observed in the TUG (*p* = 0.03), with the CG presenting better results (14.89 ± 5.29s) than the IG (21.90 ± 8.87s). No statistically significant differences were observed at baseline between groups in the SEE (*p* = 0.26) and the PAPM (*p* = 0.10).

**Figure 2:**
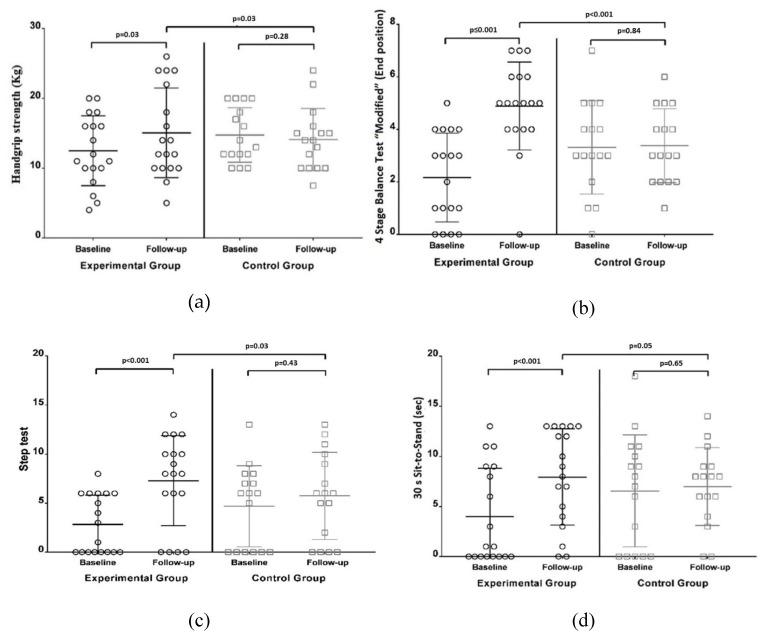

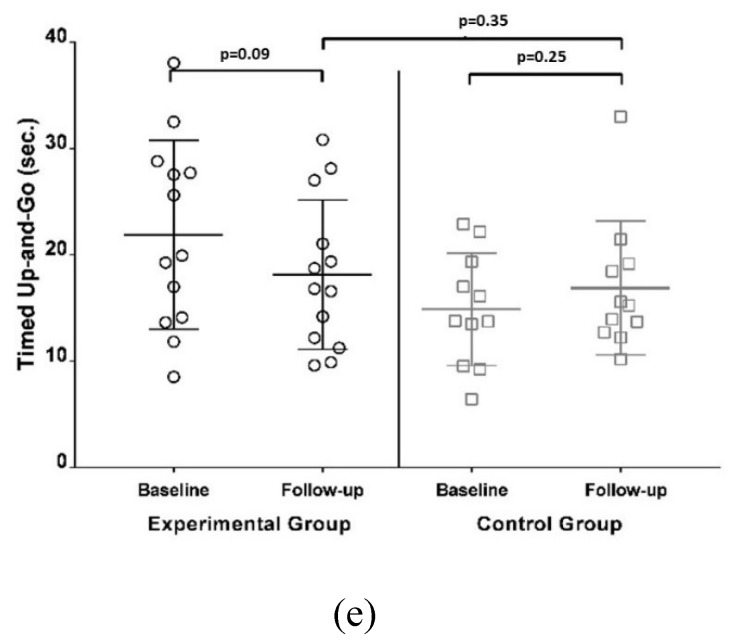
Functional ability scores in the control group and the intervention group at baseline and post intervention. (**a**): Handgrip Strength; (**b**): 4 Stage Balance Test “Modified”; (**c**): Step Test; (**d**): 30s Sit-To-Stand; (**e**) Timed Up and Go.

At the end of the 8-week program, a significant increase in HS was found in the IG (IG: 15.06 ± 6.42 Kg/F versus CG: 14.09 ± 4.46 Kg/F; *p* = 0.03) (Figure 2). No significant changes were observed in the CG. Similar results were detected in the 4StageBTM, the ST, and the 30s STS, with significant improvement observed only in the IG. No statistically significant variations were detected in the CG. After intervention, TUG also improved in the IG. No significant changes were found in the CG.

**Figure 3:**
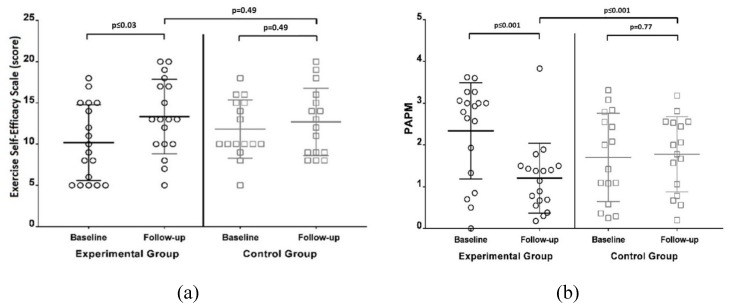
(**a**): Self-Efficacy for exercise and (**b**): social participation scores in the control group and the intervention group at baseline and post intervention. PAPM: Activities and Participation Profile related to Mobility.

The results for the SEE and the PAPM are presented in Figure 3. A significant improvement in the SEE was observed only in the IG, presenting a better mean score than the CG after the intervention (13.33 ± 4.51 points vs. 12.69 ± 4.09 points, respectively), although this was not statistically significant (*p* = 0.49). However, the differences observed in the PAPM between the IG and the CG were statistically significant, with the IG presenting better participation performance (a lower score) than the CG after the intervention period (1.20 ± 0.84 and 1.78 ± 0.90, respectively; *p* < 0.001).

## 4. Discussion

Technological interventions, namely biofeedback systems and Exergames, have recently been shown to have the ability to objectively monitor therapeutic exercise in real time, and therefore to improve performance quality [45].

The baseline assessment indicated a mild to moderate disability and a significant risk of falling among the participants: less than 10 steps [46], TUG > 10 s [43,47], HS lower than 15 kg and 21 kg, in female and male, respectively [48], an inability to keep 10 s in the tandem stance position (eyes opened) [49,50], and 30s STS < 10 male and < 9 female [51]. After the intervention, significant improvements in balance, muscular strength, gait, and social participation were reported.

In a study that compared virtual reality programs including strength, endurance, flexibility, and balance exercises with a simple balance exercise virtual reality program, the former produced better results [52]. The same effect was revealed in our study once more than one type of exercise was integrated.

A study that compared an augmented reality Otago based exercise program with a traditional Otago program demonstrated that technology added value to the program [53]. Another study that investigated the effect of an Otago program implemented via an interactive DVD for six months, with telephone monitoring by a physiotherapist once a month, also demonstrated benefits among using these positive strategies for preventing falls [54]. A balance exercise training program with the use of the Wii Fit was compared with a conventional balance exercise program, in a study which found that the Wii Fit balance training was more effective in reducing the incidence of falls in older adults with a history of falls [55]. Furthermore, the effects of balance training based on a virtual reality system (Balance Rehabilitation Unit) in balance, fear of falling, and the risk of falling in older participants with a history of falls were evaluated over six weeks (twice a week, 30 min per session), with 97% overall adherence. The program was shown to be well accepted and effective in the improvement of balance, in increasing confidence, and in the prevention of falls [56].

Although there has been a trend towards improving functional capacity and participation, these results need to be analyzed with caution due to the group selection method. Participants were all enrolled in physical activities, mainly outdoor aerobic exercise, prior to this study. The “modified” OTAGO was introduced to them as a new option for exercise. Because the authors wanted to explore the effect of the Exergames, it was decided to invite all the clients of that day care center, and the group selection depended on their willingness to experience a new exercise program. In fact, a clinically relevant improvement in the TUG was identified in the participants who had obtained the worst scores in TUG, the only outcome with a statistically significant difference between groups. However, other studies have presented conflicting evidence regarding the TUG, either showing no benefit of exercise training with the use of video games [57], or revealing significant improvements in mobility and gait [58,59].

Improvement was found in all functional parameters in the IG, whilst the maintenance of balance and a trend for a decrease in muscle strength and mobility in the CG are in line with previous research [17,60], which has reported that active older adults, compared to sedentary individuals, tend to present better functional ability and a reduced risk of falls, since exercise is beneficial in the prevention of falls, functioning, and also in promoting a better quality of life.

Additionally, the IG showed an improvement in social participation from baseline to post-intervention. This may have been due to the existence of a strong motivational component in this type of intervention, which may also improve adherence. The benefits of intervention programs incorporating interactive games and biofeedback was associated with motivation, as has already been documented in previous research combining technology and exercise [61]. Most of the Exergames studies also show promising results regarding enhanced social well-being, such as reduced feelings of loneliness, increased social connection, and positive attitudes towards others [62].

The positive changes in self-efficacy for exercise may also be associated with the motivational aspect generated by the type of training used in the present study, since a prior study which used conventional exercise training (strength training, balance exercises, walking, and stair climbing) to evaluate their effects on bone mineral density, balance, strength, and self-efficacy in elderly women over 32 weeks produced no significant differences in self-efficacy in either group (training group or sedentary group) [63]. Since previous studies have shown that physically active elderly people tend to present a lower fear of falling, better balance, and a greater perception of self-efficacy [64], our results encourage physiotherapists to explore self-efficacy strategies in their daily routines with older adults.

Recently, a systematic review of “modified” OTAGO formats identified exercise programs associated with vestibular or multisensory balance exercises, the use of augmented reality, and exercise in group with a physiotherapist or with DVD support, and checked their effects on balance. The authors of the review concluded that, in general, all studies using a “modified” OTAGO reported improvements in balance and functional abilities in general. However, whether or not these adapted formats are as effective as the original OTAGO, and which is the most effective, remains unclear, because different adaptations of the OTAGO were applied in different studies [65]. In this sense, more studies are needed to bring greater clarity to this still controversial topic.

This study provides an additional scientific contribution to the potential usefulness of exercise programs incorporated in technological tools, which, in the present study, were associated with significant improvements in balance, muscular strength, mobility, self-efficacy for exercise, and social participation in older adults who voluntarily decided to integrate this “modified” OTAGO. Additional studies are needed to identify the long-term benefits, dose-dependent effects, and the most appropriate eligibility criteria and selection method to overcome the limitations which can be attributed to this study.

## 5. Conclusions

The intervention program tested in the present study, as part of an integrated approach to promote active and healthy ageing, the AGA@4life model, contributed to enhancing the functional abilities and social participation of the older adults, promoted their self-efficacy in relation to exercise, and reached a high level of adherence, all factors that are associated with falls prevention. These effects are even more promising if we consider that the older adults who were not enrolled in this program showed a more marked decline of their overall functional parameters. The AGA@4life model thus seems to be an effective approach to the promotion of wellness, autonomy, and health in a progressively older population.

## Figures and Tables

**Table 1:** Demographic and clinical characterization of the total sample, control group, and intervention group at baseline.

Variables	Total Sample (*n* = 34)	Control Group (*n* = 16)	Intervention Group (*n* = 18)	*p*-Value
Age, years	83.24 ± 6.89	84.88 ± 7.27	83.06 ± 8.52	0.763
Gender				
Female, % (*n*)	23.53 (8)	81.25 (13)	72.22(13)	0.690
Male, % (*n*)	76.47(26)	18.75 (3)	27.78 (5)	
History of falls in the last 12 months				
Yes, % (*n*)	41.18 (14)	31.25 (5)	50.00 (9)	0.320
No, % (*n*)	58.82 (20)	68.75 (11)	50.00 (9)	
Fear of falling				
Yes, % (*n*)	85.29 (29)	81.25 (13)	88.89 (16)	0.650
No, % (*n*)	14.71 (5)	18.75 (3)	11.11 (2)	
Sedentary lifestyle				
Yes, % (n)	76.47 (26)	75.00 (12)	77.78 (14)	1.00
No, % (n)	23.53 (8)	25.00 (4)	22.22 (4)	
Upper extremities assistance to stand from a chair				
Yes, % (n)	73.53 (25)	62.50 (10)	83.33 (15)	0.250
No, % (n)	26.47 (9)	37.50 (6)	16.67 (3)	
